# SRC-like adaptor protein 2 (SLAP2) is a negative regulator of KIT-D816V-mediated oncogenic transformation

**DOI:** 10.1038/s41598-018-24743-y

**Published:** 2018-04-23

**Authors:** Kaja Rupar, Sausan A. Moharram, Julhash U. Kazi, Lars Rönnstrand

**Affiliations:** 1 Division of Translational Cancer Research, Department of Laboratory Medicine, Lund University, Lund, Sweden; 2 Lund Stem Cell Center, Department of Laboratory Medicine, Lund University, Lund, Sweden; 3 Division of Oncology, Skåne University Hospital, Lund, Sweden

## Abstract

KIT is a receptor tyrosine kinase (RTK) involved in several cellular processes such as regulation of proliferation, survival and differentiation of early hematopoietic cells, germ cells and melanocytes. Activation of KIT results in phosphorylation of tyrosine residues in the receptor, and recruitment of proteins that mediate downstream signaling and also modulate receptor signaling. Here we show that the SRC-like adaptor protein 2 (SLAP2) binds to wild-type KIT in a ligand-dependent manner and is furthermore found constitutively associated with the oncogenic mutant KIT-D816V. Peptide fishing analysis mapped pY568 and pY570 as potential SLAP2 association sites in KIT, which overlaps with the SRC binding sites in KIT. Expression of SLAP2 in cells expressing the transforming mutant KIT-D816V led to reduced cell viability and reduced colony formation. SLAP2 also partially blocked phosphorylation of several signal transduction molecules downstream of KIT such as AKT, ERK, p38 and STAT3. Finally, SLAP2 expression enhanced ubiquitination of KIT and its subsequent degradation. Taken together, our data demonstrate that SLAP2 negatively modulates KIT-D816V-mediated transformation by enhancing degradation of the receptor.

## Introduction

The stem cell factor (SCF) receptor, KIT, is a type III receptor tyrosine kinase (RTK) which regulates differentiation, proliferation and migration of early hematopoietic cells, germ cells and melanocytes and is expressed in wide range of cell types. Wild-type KIT is activated upon binding of its ligand, stem cell factor (SCF), which leads to receptor dimerization, activation of its intrinsic tyrosine kinase activity followed by autophosphorylation of KIT. Binding of SRC Homology 2 (SH2) domain-containing proteins to phosphotyrosine residues in KIT do either positively or negatively regulate downstream signaling. Oncogenic mutations, that are found in KIT in many types of cancer and leukemia, result in dysregulated KIT activation and thus aberrant activation of downstream signaling^1^. The most frequently found oncogenic KIT mutation, D816V^1^, causes constitutive and SCF-independent activation of the receptor^2^. Receptor-mediated signals need to be tightly regulated and modulated in order to prevent persistent signaling under normal physiological conditions. The activity of KIT can be negatively regulated by several different mechanisms, such as protein tyrosine phosphatases that dephosphorylate the receptor or downstream targets, as well as ubiquitin-mediated degradation of the activated receptor. Here we show that the SRC-like adaptor protein 2 (SLAP2) regulates KIT stability and downstream signaling by promoting ubiquitination of KIT and its subsequent degradation.

SLAP2 is an adaptor protein involved in the regulation of multiple signaling pathways^3^, (reviewed by^4^). It is expressed in several hematopoietic cell types including stem cells, platelets, monocytes, macrophages and T- and B-cells. In humans, SLAP2 is a 261 amino acid long protein encoded by the *SLA2* gene which is localized to chromosome 20q11.23. SLAP2 is a close homolog of SLAP and its structure is similar to that of the SRC family kinases (SFKs). It consists of an amino-terminal region, a SRC Homology 3 (SH3) domain, a SRC Homology 2 (SH2) domain and a carboxy-terminal region, but in contrast to the SRC family members, it lacks kinase activity. The amino-terminal region can undergo posttranslational myristoylation, which enables SLAP2 to associate with the cell membrane, while the non-myristoylated SLAP2 is localized to the nucleus^5^. The SLAP2 SH3 domain interacts with proline-rich sequences in proteins and thus mediates protein-protein interactions that regulate intracellular signal transduction pathways. The SH2 domain is necessary for binding to phosphorylated tyrosine residues in activated receptor tyrosine kinases and other tyrosine phosphorylated proteins. In contrast to many other adapter proteins containing both SH2 and SH3 domains, the SH3 and SH2 domains of SLAP2 adaptor protein interact with one another in an alternative mode that leads to the formation of a beta-sheet comprised of both domains. The functional integrity of both the SH2 and the SH3 domains is maintained in this structure^6^. Finally, the carboxy-terminal region mediates SLAP2 association with the ubiquitin E3 ligase CBL (Casitas B-lineage Lymphoma)^5^. SRC-like adaptor proteins are well established as negative regulators of T-cell receptor signaling^3,7^ and recent studies also implicate their negative role in receptor tyrosine kinase signaling by promoting ubiquitin-mediated receptor tyrosine kinase degradation^8^. Specifically, a study from 2007 showed that SLAP2 negatively regulates signaling through the type III receptor tyrosine kinase colony-stimulating factor-1 receptor (CSF1R) by recruiting CBL to the activated receptor, which results in enhanced ubiquitination and degradation of the receptor^9^. Furthermore, we have recently shown that SLAP2 binds to and negatively regulates another type III receptor tyrosine kinase, Fms like tyrosine kinase 3, FLT3^10^. Therefore, we hypothesized that SLAP2 might play a role in the RTK KIT. We here show that SLAP2 binds to wild-type KIT in response to SCF stimulation and is constitutively associated with the oncogenic mutant KIT-D816V. The association is mediated through the SH2 domain of SLAP2. Association of SLAP2 with KIT results in negative regulation of KIT downstream signaling.

## Results

### SLAP2 associates in a ligand-dependent manner with KIT through its SH2 domain

**Figure 1:**
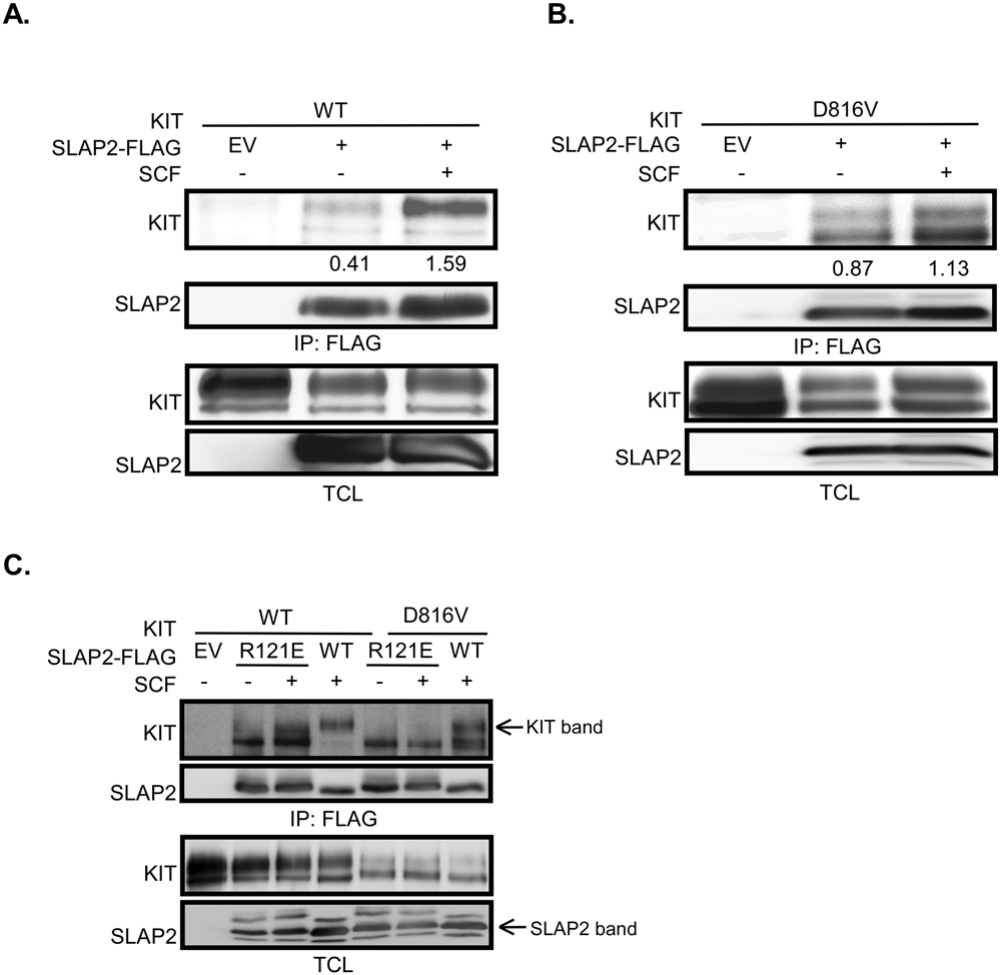
SLAP2 associates with KIT via its SH2 domain: (**A**) COS-1 cells were co-transfected with plasmids carrying wild-type KIT and SLAP2-WT-FLAG or an empty vector (EV). 24 hrs after transfection, cells were stimulated with 100 ng/ml of SCF for 5 min, lysed and immunoprecipitated with anti-FLAG antibody, and thereafter analyzed by Western blotting using specific antibodies. KIT shows up as two bands in the cell lysate, while upper band was immunoprecipitated with SLAP2. (**B**) COS-1 cells were co-transfected with KIT-D816V plasmid and a plasmid carrying SLAP2-WT-FLAG or an EV. One day after transfection, cells were stimulated for 5 min with 100 ng/ml SCF and lysed. Cell lysates were immunoprecipitated with anti-FLAG antibody and were further analyzed by Western blotting. (**C**) COS-1 cells were transfected with wild-type KIT or KIT-D816V and SLAP2-WT-FLAG or SLAP2-R121E-FLAG plasmids or an EV. 24-hours after transfection, cells were stimulated for 5 min with 100 ng/ml SCF and lysed. Anti-FLAG antibody was used to immunoprecipitate cell lysates and cells where further subjected to Western blot analysis. EV: empty vector, IP: immunoprecipitation, TCL: total cell lysate. KIT is displayed as two bands in Western blotting. The upper band represent the fully glycosylated proteins while the lower band is the immature, partially glycosylated proteins. We have repeatedly seen three bands in total cell lysates with anti-FLAG antibody. While the strong band in the middle represents the SLAP2-FLAG band, the other two faint bands might be nonspecific bands. The blots were cropped to focus upon the specific proteins indicated.

A recent study has shown that SLAP, a close homolog of SLAP2, associates with wild-type KIT as well as the oncogenic mutant KIT-D816V^11^. Since SLAP2 has also been shown to interact with other type III RTKs such as CSF1R^9^ and FLT3^10^, we sought to investigate the possible role of SLAP2 in KIT signaling. In order to determine whether SLAP2 can interact with KIT, we overexpressed FLAG-tagged SLAP2 and wild-type KIT in COS-1 cells and incubated cells in the absence or presence of SCF for 5 min. Immunoprecipitation using an anti-FLAG antibody directed against SLAP2 showed that SLAP2 only associates with wild-type KIT following ligand-stimulation (Fig. 1A). The upper band of KIT is highly glycosylated, is localized to the cell surface and responds to the SCF stimulation. In order to determine whether SLAP2 also interacts with an oncogenic KIT mutant (KIT-D816V), COS-1 cells were co-transfected with KIT-D816V and SLAP2-FLAG and incubated in the presence or absence of stimulation with SCF for 5 min. We observed constitutive association of SLAP2 with KIT-D816V (Fig. 1B). The KIT-D816V mutant is constitutively active in a ligand-independent manner^2^ which explains why SLAP2 interaction with KIT-D816V was independent of SCF stimulation. Since the interaction between SLAP2 and wild-type KIT is dependent on phosphorylation of KIT on tyrosine residues, it is likely that the SH2 domain of SLAP2 is involved in the interaction. Furthermore, it has previously been shown that the closely related protein SLAP associates with KIT through its SH2 domain^11^. In order to investigate the possible role of the SH2 domain, we generated a mutant of the SLAP2 SH2 domain that fails to bind phosphotyrosine (SLAP2-R121E-FLAG). In this mutant, the positively charged arginine residue in the phosphotyrosine binding pocket of the SH2 domain was replaced by a negatively charged glutamic acid, thus blocking binding to phosphotyrosine residues^10^. COS-1 cells were co-transfected with either wild-type KIT or KIT-D816V and wild-type SLAP2 or SLAP2-R121E or an empty vector (EV) and incubated in the absence or presence of SCF for 5 min. Immunoprecipitation showed that only wild-type SLAP2 was able to interact with SCF-stimulated wild-type KIT and KIT-D816V, while the interaction was inhibited in the SLAP2 SH2 domain mutant (Fig. 1C). These data demonstrate that SLAP2 associates with SCF-stimulated wild-type KIT, while the interaction of SLAP2 with KIT-D816V is ligand-independent, but that SH2 domain is necessary for association of SLAP2 with either receptor type.

### SLAP2 associates mainly through pY568 and pY570 in KIT

**Figure 2:**
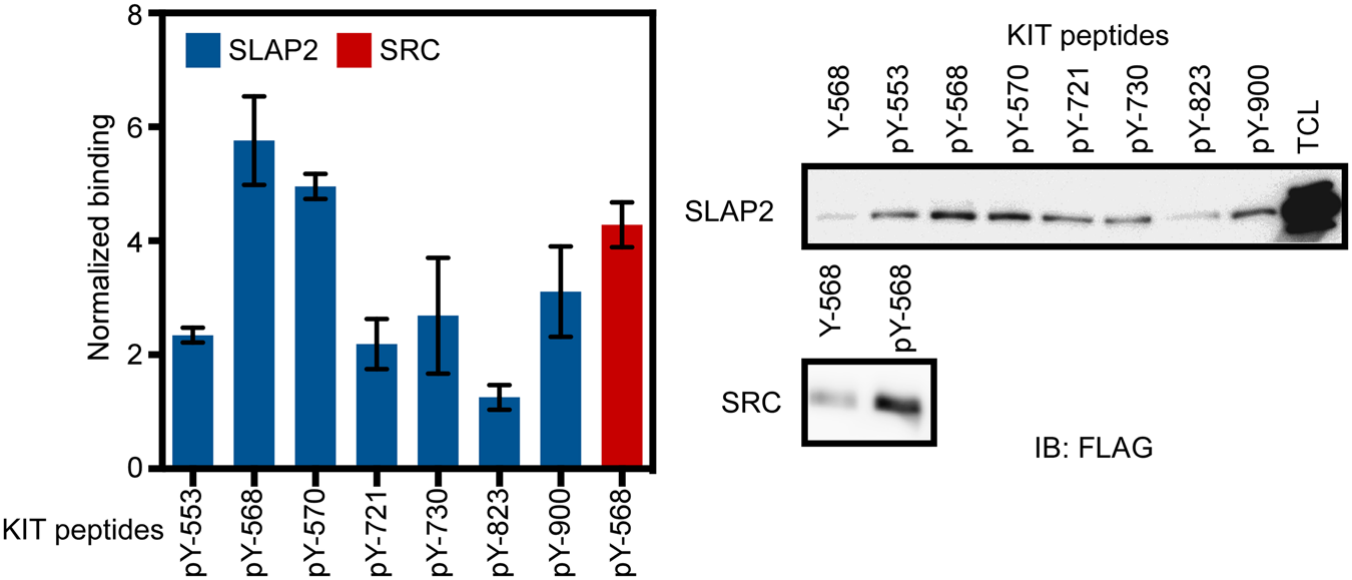
SLAP2 associates with KIT mainly via the pY568 and pY570 residues in KIT: COS-1 cells were transfected with SLAP2-FLAG plasmid. Cells were lysed 24 hours after transfection and lysates were incubated with immobilized phosphopeptides, corresponding to known KIT tyrosine phosphorylation sites. A corresponding non-phosphorylated peptide was used as control. After washing the beads four times, samples were analyzed by Western blotting. Blots of four experiments were quantified. SRC-FLAG was used as a control. TCL: total cell lysate, IB: immunoblot, pY: phosphotyrosine residue. SLAP2 protein associating withKIT phosphopeptides was normalized against proteins associating with the corresponding non-phosphorylated peptid, Y-570. The experiment was repeated three times and the error bar shows SEM. The blots were cropped to focus upon the specific proteins indicated.

In order to map the SLAP2 binding sites in KIT, synthetic phosphopeptides corresponding to known KIT tyrosine phosphorylation sites were used. A non-phosphorylated peptide corresponding to Y568 was used as a control and for normalization. Binding of SRC to the pY568 residue was used as a positive control^12^. The results demonstrate that SLAP2 association with KIT mainly occurs through two phosphotyrosine residues, pY568 and pY570, located in the juxtamembrane region of the receptor (Fig. 2).

### SLAP2 reduces KIT-D816V-mediated cell viability

**Figure 3:**
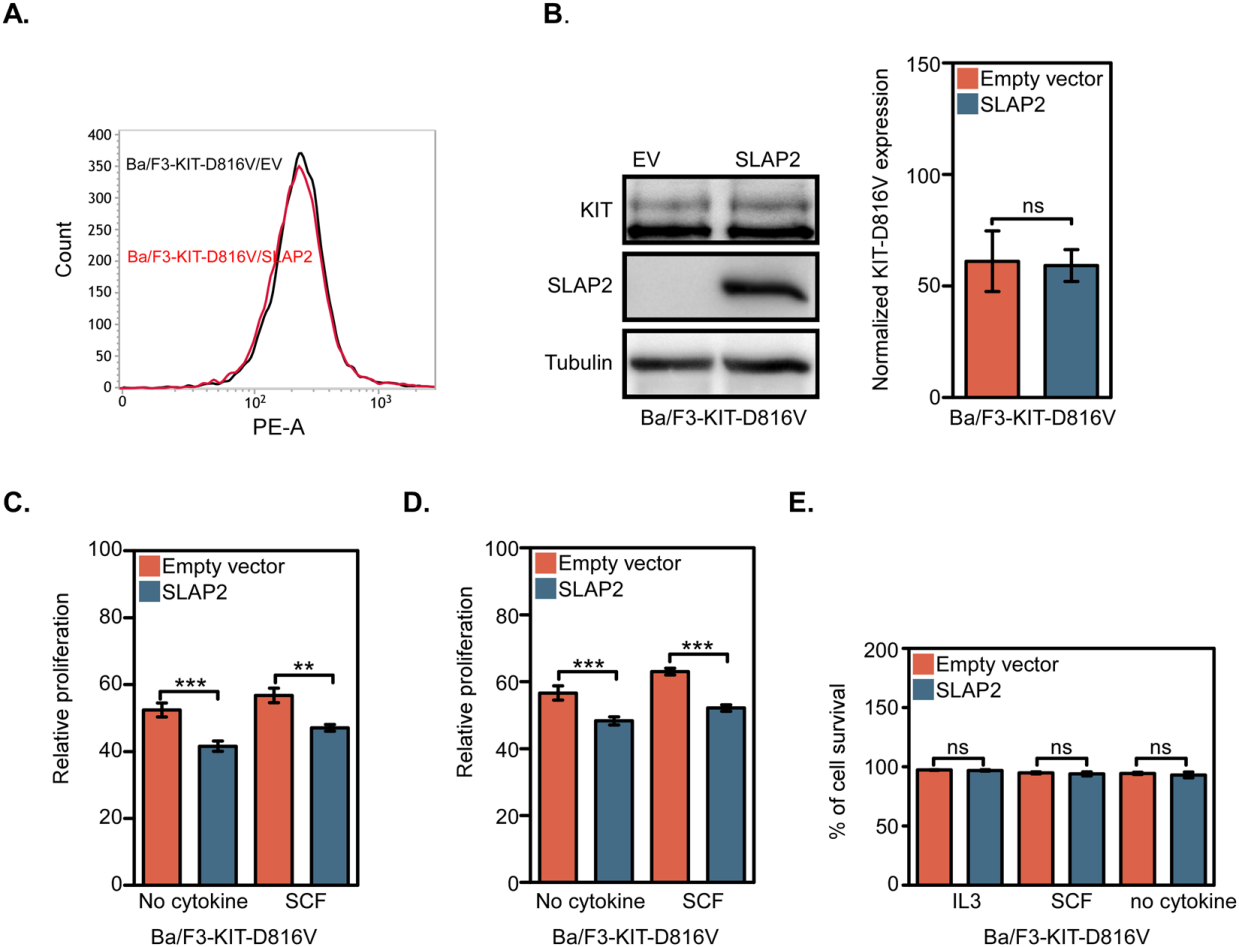
SLAP2 negatively regulates KIT-D816V-mediated cell proliferation but not cell survival: (**A**) Ba/F3 cell line stably transfected with KIT-D816V and SLAP2-WT or an EV was generated using retroviral transfection. Cells were labelled using PE anti-human CD117 or an isotype control. KIT-D816V surface expression was measured with flow cytometry. (**B**) After cells stably transfected with KIT-D816V and SLAP2 or an EV were lysed, the total amount of SLAP2 and KIT-D816V expressed was determined with Western blot. For quantification of the data, blots form three independent experiments were used. Normalization of KIT expression was performed against tubulin expression. Arbitrary units were used to generate the bar graph. The blots were cropped to focus upon the specific proteins indicated (**C**,**D**) Cells were washed and seeded on 96-well plates with IL-3 or with SCF or without IL-3 and SCF. After 48 hour of incubation, either CellTiterGlo Reagent (**C**) or PrestoBlue (**D**) was added to the cells and luminescence or fluorescence, respectively, was measured. Relative viability of the cells was calculated by normalizing the data against viability of cells, cultured in the presence of IL-3. (**E**) Cells were washed, seeded in 12-well plates and incubated for 48 hours in presence of IL-3 or SCF or without cytokine. Cells were then stained with fluorescent Annexin V antibody and % of cell survival was determined using flow cytometry. **P ≤ 0.01, ***P ≤ 0.001 ns, non-significant.

KIT is involved in controlling different cellular processes such as differentiation, proliferation and migration. Therefore, in order to investigate the biological function of the SLAP2/KIT interaction, Ba/F3 cells, which lack endogenous KIT expression^13^, were stably transfected with KIT-D816V and SLAP2 or empty vector. The expression of SLAP2 in Ba/F3 cells has not been determined due to a lack of working antibodies against SLAP2. Ba/F3 cells are dependent on murine IL-3. Cell surface expression of KIT-D816V was measured by flow cytometry and showed that KIT-D816V is expressed to an equal level in Ba/F3 cells co-transfected with either SLAP2 or an empty vector (Fig. 3A). The total KIT-D816V and SLAP2 expression was determined by Western blotting (Fig. 3B). Relative cell viability was measured using two independent methods which both showed that cell viability is decreased by SLAP2 expression (Fig. 3C,D). However, SLAP2 did not display any effect on apoptosis (Fig. 3E). Thus, we conclude that SLAP2 reduces KIT-D816V-mediated cell viability without inducing apoptosis.

### SLAP2 is a negative regulator of KIT-D816V-mediated *in vitro* colony formation

**Figure 4:**
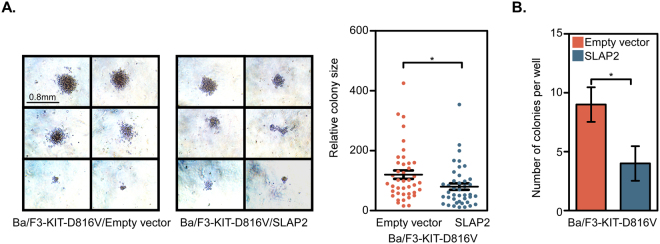
SLAP2 negatively regulates KIT-D816V-mediated colony formation: (**A**,**B**) Ba/F3 cells expressing KIT-D816V were washed three times to remove serum and cytokines prior to seeding in a 24-well plate at a concentration of 500 cells per well. Cells were grown in a methylcellulose medium for 7 days. *P ≤ 0.05. Colonies were counted by two individual investigators. Two biological replicates and five technical replicates were used.

In order to assess the influence of SLAP2 on colony forming ability of KIT-D816V expressing cells, Ba/F3 cell line expressing KIT-D816V, together with SLAP2 or empty vector, were grown for 7 days on methylcellulose medium. The relative colony size of cells expressing SLAP2/KIT-D816V was significantly smaller compared to empty vector control/KIT-D816V expressing cells (Fig. 4A). The number of colonies per well was also significantly reduced in SLAP2 expressing cells (Fig. 4B). Thus, in conclusion, SLAP2 decreases the KIT-D816V-mediated clonogenic potential.

### SLAP2 is a negative regulator of signaling pathways downstream of KIT

**Figure 5:**
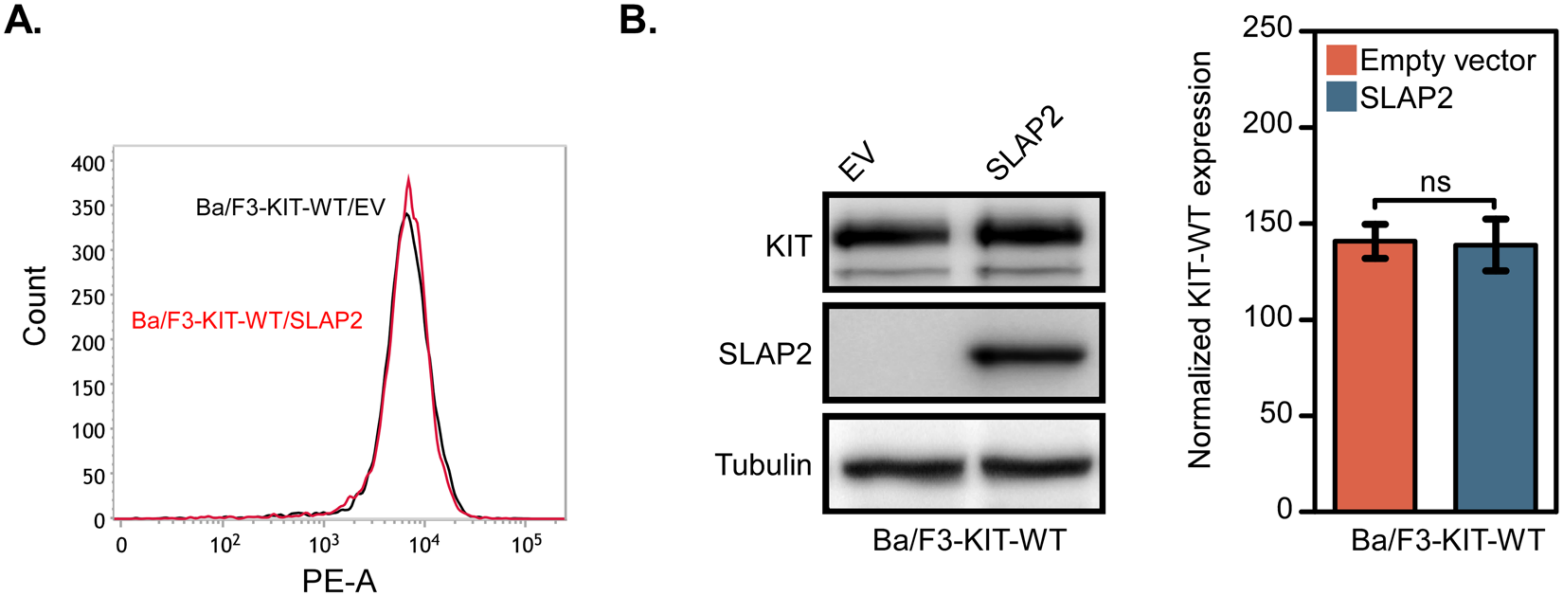
KIT and SLAP2 expression in Ba/F3 cell lines: (**A**) Ba/F3 cells with stable expression of wild-type KIT and SLAP2 or an EV were created by retroviral transfection. Cells were labelled with PE anti-human CD117 or an isotype control. The surface expression of wild-type KIT was determined using flow cytometry. (**B**) Ba/F3-KIT-WT/EV and Ba/F3-KIT-WT/SLAP2 cells were lysed and the total amount of KIT and SLAP2 was determined by Western blotting. Blots form three independent experiments were used for quantification of the data. Normalization of KIT expression was performed against tubulin expression. Arbitrary units were used to generate the bar graph. ns, non-significant. The blots were cropped to focus upon the specific proteins indicated.

**Figure 6:**
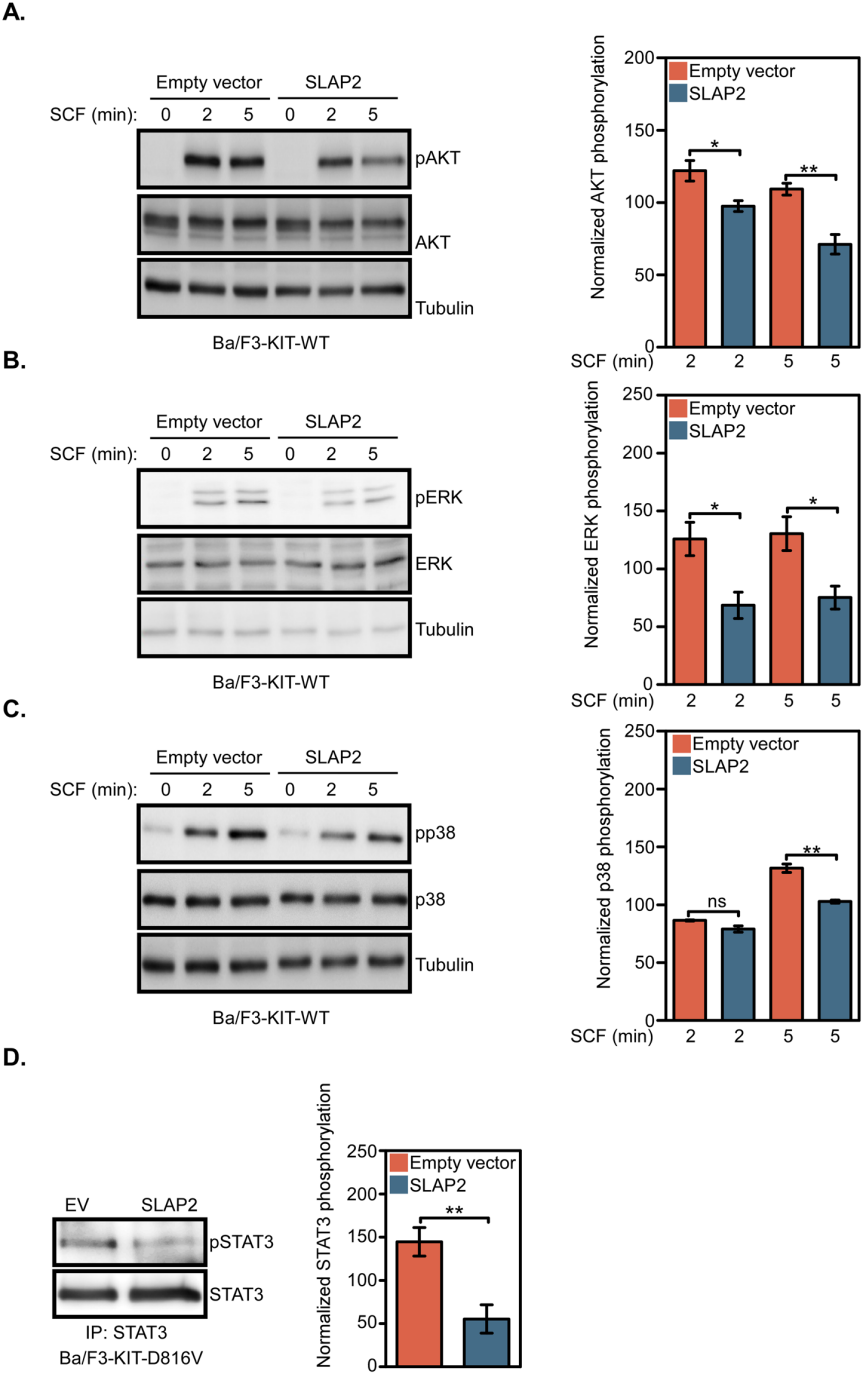
Signaling downstream of KIT is partially blocked by SLAP2: (**A**–**C**) Ba/F3 cells expressing wild-type KIT were washed three times and serum- and IL-3-starved for 4 hours. Prior to cell lysis, cells were stimulated with 100 ng/ml SCF for 0, 2 and 5 min. Cell lysates were analyzed by Western blotting and blots of multiple experiments were quantified to determine statistical significance. (**D**) Ba/F3 cells expressing KIT-D816V were washed three times and then serum- and cytokine-starved for 4 hours. Cells were lysed and subjected to immunoprecipitation with an anti-STAT3 antibody. Statistical analysis was performed by quantifying Western blots from three independent experiments. Normalization of phospho-proteins was performed against total protein. IP, immunoprecipitation, *P ≤ 0.05, **P ≤ 0.01, ns: non-significant. The blots were cropped to focus upon the specific proteins indicated.

In order to understand how SLAP2 negatively regulates KIT-mediated cell proliferation, we generated Ba/F3 cells stably expressing wild-type KIT together with or without SLAP2. The cell surface expression of KIT in cells expressing SLAP2 or not was determined by flow cytometry (Fig. 5A) and the total expression of KIT and SLAP2 in these cells was measured using Western blotting (Fig. 5B). Upon IL-3 withdrawal, Ba/F3 cells transfected with wild-type KIT respond to the SCF stimulation. Activation of wild-type KIT induces activation of the PI3K/AKT, MAPK-ERK and p38 pathways^1^. It has also been shown that KIT mediates phosphorylation and activation of transcription factors of the STAT family^14–16^. Thus, determination of the phosphorylation status of signal transduction molecules downstream of KIT enabled us to investigate the influence SLAP2 has on signaling downstream of KIT. Western blot analysis showed that SLAP2 significantly decreased AKT and ERK phosphorylation after 2 and 5 min of stimulation with SCF, while p38 phosphorylation was significantly decreased after 5 min of SCF stimulation (Fig. 6A–C). Previous studies have demonstrated a dependency of STAT3 signaling for KIT-D816V-induced transformation^17,18^. We could also see a significant decrease in STAT3 phosphorylation in Ba/F3 cells overexpressing KIT-D816V and SLAP2, compared to cells only expressing KIT-D816V (Fig. 6D**)**. In summary, these data suggest that SLAP2 negatively regulates KIT signaling by decreasing the phosphorylation of signal transduction molecules downstream of the receptor.

### SLAP2 expression enhances ubiquitin-mediated KIT degradation

**Figure 7:**
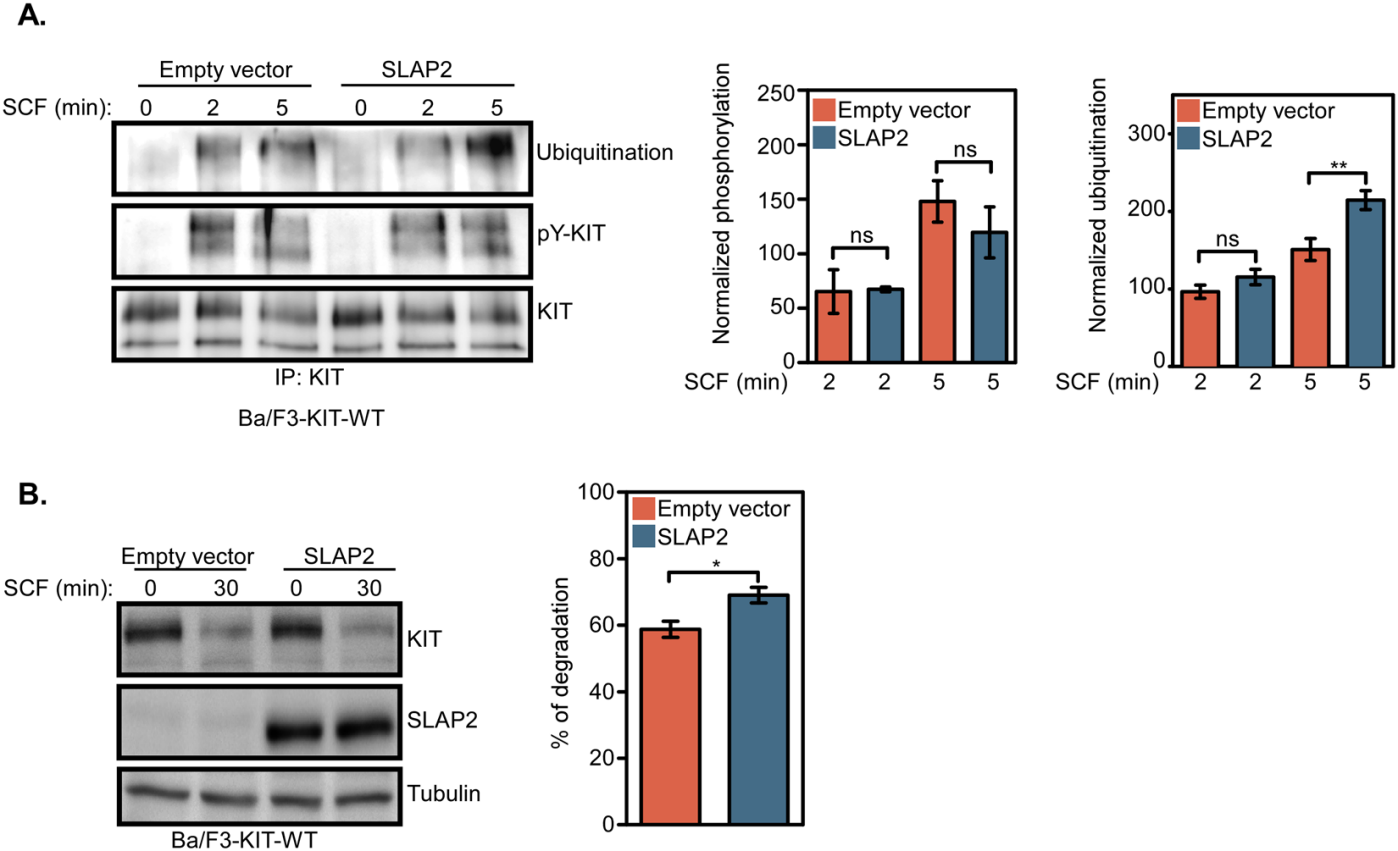
SLAP2 facilitates ubiquitin-mediated KIT degradation: (**A**) Ba/F3 cells co-expressing wild-type KIT and SLAP2 or an EV were washed three times before it was serum- and IL-3-starved for 4 hours. Prior to cell lysis, cells were stimulated with SCF for 0, 2 and 5 min. Cell lysates were immunoprecipitated with an anti-KIT antibody and analyzed by Western blotting. Blots from multiple experiments were quantified for statistical analysis. Total FLT3 tyrosine phosphorylation was detected by using 4G10 anti-phospho-tyrosine antibody. Ubiquitination and total tyrosine phosphorylation was normalized against total FLT3. (**B**) Ba/F3 cells co-expressing wild-type KIT and SLAP2 or an EV were washed three times and treated with cycloheximide for 30 min at 37 °C. Cells were SCF stimulated for 30 min, lysed and lysates were subjected to Western blot analysis. Quantification of blots from three independent experiment was performed. IP: immunoprecipitation, TCL: total cell lysate, pY: phosphotyrosine residue, *P ≤ 0.05, **P ≤ 0.01, ns: non-significant. The blots were cropped to focus upon the specific proteins indicated.

The signal transduction processes downstream of activated RTKs need to be modulated so that there is not to strong or to weak signal. It is known that too strong RTK signaling can lead to induction of apoptosis^19,20^. Thus, the activity of proteins involved in cellular signal transduction processes must be negatively regulated either by dephosphorylation through the action protein tyrosine phosphatases or degraded through ubiquitin-mediated proteasomal or lysosomal degradation. It has previously been reported that SLAP2 downregulates the CSF1R by recruiting the ubiquitin E3 ligase CBL to the receptor^9^ which leads to increased ubiquitination which targets the receptor for degradation. To investigate whether SLAP2 regulates KIT ubiquitination and stability, Ba/F3 cells expressing wild-type KIT were stimulated with SCF for 0, 2 or 5 min. Cell lysates were subjected to immunoprecipitation using an anti-KIT antibody and analyzed for tyrosine phosphorylation by Western blotting. The data show that KIT phosphorylation remained almost unaffected by SLAP2 expression, while receptor ubiquitination was significantly increased (Fig. 7A). In order to determine whether SLAP2 affects the rate of KIT degradation, Ba/F3 cells expressing wild-type KIT were treated with cycloheximide (an inhibitor of protein synthesis) for 30 min prior to stimulation with SCF for 30 min. Cells were then lysed and analyzed by Western blotting. Consistent with our previous results that demonstrated SLAP2-mediated increase in KIT ubiquitination, the quantified blots demonstrate a significantly higher rate of receptor degradation in cells overexpressing SLAP2 compared to the control (Fig. 7B). In conclusion, our data demonstrate that expression of SLAP2 enhances ubiquitin-mediated degradation of KIT which could explain the effects on cellular signaling.

## Discussion

By regulating several signaling pathways, RTKs are involved in a variety of cellular processes and therefore the maintenance of a balance between activation of positive and negative signals and the termination of signals is of great importance. Aberrations in signal transduction are associated with a number of diseases, including cancer, diabetes etc. In this study we identify SLAP2 as a novel KIT interacting protein, which regulates receptor signaling by reducing the activity of its downstream signal transduction targets and by promoting degradation of the receptor. Furthermore, we show that SLAP2 is a negative regulator of KIT-D816V-mediated transformation. Negative regulators might, in a somewhat contradictory manner, be of importance for transformation. The tumor cells need a precise level of proliferative signals and survival signals. Too much or too little will induce apoptosis, which is why the cells need to be able to modulate the levels of signals^19,20^. It is well known that a number of oncogenic mutants of RTKs indeed upregulate the expression of negative regulators such as SOCS proteins and that e. g. leukemic cells express high levels of SOCS proteins than their normal cellular counterparts^21^.

A recent study showed that SLAP, a close homolog of SLAP2, interacts with and regulates signaling downstream of KIT^11^. Although SLAP2 expression overlaps with that of SLAP, it has been shown that the two proteins don’t function in an identical manner in relation to signaling downstream of RTKs^8,10,22^. For instance, while depletion of SLAP using shRNA resulted in negative regulation of FLT3 downstream signaling, overexpression of SLAP2 led to a similar response indicating that SLAP and SLAP2 play opposite roles in regulation of FLT3 downstream signaling. It has been shown that the wild-type receptor requires activation by its ligand, SCF, in order to be able to associate with SLAP^11^. However, the constitutively active KIT-D816V mutant associates with SLAP independent of ligand-stimulation^11^. Our data demonstrate that association of SLAP2 with the receptor occurs via its SH2 domain. We were also able to identify two phosphotyrosine residues in KIT, pY568 and pY570, that are involved in SLAP2 association. Interestingly, those sites are also binding sites for SFKs^12^, raising the possibility that SLAP2 competes with SFKs for association with KIT. However, there are many additional signal transduction proteins that have been demonstrated to associate with pY568 (CHK, LNK, PTPN11, APS, CBL) demonstrating that SFKs are not the only candidate proteins that SLAP2 might compete with for binding^23–27^.

The data presented in this study suggest that SLAP2 is a modulator of KIT-mediated signaling. Activation of KIT signaling results in activation of several signaling cascades including the PI3K/AKT, MAPK/ERK and p38 pathways. The signals mediated by oncogenic mutants of KIT are by and large the same as those activated by wild-type KIT, but in addition, KIT-D816V activates STAT5 signaling^28^. Aberrant activation of STAT proteins can lead to tumorigenic cell transformation^29^. Therefore, it is likely that SLAP2 controls the colony forming capacity of KIT-D816V through negative regulation of STAT3 phosphorylation. However, since SLAP2 expression resulted in negative regulation of AKT, ERK, p38 and STAT3 phosphorylation, there is no detailed selectivity in terms of inhibition of signaling, but rather that the effect goes through degradation of the receptor. This is also supported by the observed SLAP2-dependent enhancement of KIT ubiquitination, leading to accelerated KIT degradation. Since binding sites for SLAP2 overlap with those of SFK, it is also possible that SLAP2 competes with SFK for association to KIT and thereby, in addition, inhibits SRC-mediated signaling. Ubiquitin-mediated proteasomal or lysosomal degradation of activated RTKs is an important mechanism by which cells control receptor signaling^30^. The viral form of KIT, v-KIT, displays a deletion of the amino acids involved in binding of CBL to the receptor, leading to increased stabilization of the receptor and thereby enhanced signaling^31^. It has previously been reported that SLAP2 facilitates CSF-1R ubiquitination and degradation by recruiting the ubiquitin E3 ligase CBL to the receptor^9^. SLAP also play a role in KIT and FLT3 ubiquitination^11,22^. Our data demonstrates that SLAP2 significantly promotes ubiquitination and subsequent degradation of KIT, without altering the receptor tyrosine phosphorylation. The ubiquitin E3 ligase CBL has the ability to associate with activated KIT directly or indirectly via adaptor proteins such as GRB2, APS and CRKL^26,27,32,33^. Therefore, all these proteins, including SLAP2, contribute to ubiquitination and degradation of SCF-activated KIT in order to maintain a physiological balance of receptor signaling.

Taken together, our data demonstrate that SLAP2 acts as a negative regulator of KIT signaling and that expression of SLAP2 partially represses KIT-D816V-mediated oncogenesis.

## Materials and Methods

### Reagents, antibodies and plasmids

The luminescent cell viability assay kit, CellTiter-Glo was from Promega (Madison, WI). Cycloheximide, horseradish peroxidase-coupled anti-FLAG (M2) and mouse anti-FLAG (M2) were from Sigma-Aldrich (St Louis, MI). Dulbecco’s Phosphate-Buffered Saline (DPBS), Dulbecco’s Modified Eagle’s Medium (DMEM), RPMI 1640 medium and Penicillin/ Streptomycin solution were from Corning (Corning, NY). Human recombinant stem cell factor (SCF) and murine recombinant interleukin-3 (IL-3) were from ProSpec Tany Technogene (Ness-Ziona, Israel). Iscove’s Modified Dulbecco’s Medium (IMDM), Lipofectamine 2000, PrestoBlue Cell Viability Reagent, Heat-inactivated fetal bovine serum (FBS), UltraLink beads, horseradish peroxidase-coupled secondary anti-mouse and anti-rabbit antibodies were from ThermoFisher Scientific (Waltham, MA). Methylcellulose medium and PE Annexin V Apoptosis Detection Kit were from Stem Cell Technologies (Vancouver, Canada) and BD Biosciences (Franklin Lakes, NJ), respectively. Goat anti-AKT antibody, horseradish peroxidase-coupled secondary anti-goat antibody, rabbit anti-ERK2, rabbit anti-KIT antibody, rabbit anti-phospho-ERK1/2 (pThr202/pThr204) antibody and rabbit anti-STAT3 were from Santa Cruz Biotechnology (Santa Cruz, CA). Mouse anti-mono-ubiquitin antibody, mouse anti-phosphotyrosine (4G10) antibody and rabbit anti-phospho- AKT (pSer473) antibody were from Covance Research Products (Princeton, NJ), Merck Millipore (Billerica, MA) and Abcam (Cambridge, UK), respectively. Mouse anti-phospho-p38 and anti-p38 antibodies were from BD Biosciences (Sparks, MD). PE-conjugated anti-human CD117 and PE-conjugated mouse IgG1 isotype control antibodies were from BioLegend (San Diego, CA). Plasmids encoding wild-type KIT (pcDNA3-KIT-WT^34^ and pMSCV-KIT-WT^35^) KIT-D816V (pcDNA3-KIT-D816V and pMSCV-KIT-D816V^36^), wild-type SLAP2 and the SLAP2 SH2 domain mutant (pMCV-SLAP2-WT-Myc-FLAG and pCMV-SLAP2-R121E-Myc-FLAG^10^) were previously described.

### Cell culture

COS-1 cells were cultured in DMEM supplemented with 1% Penicillin/Streptomycin solution and 10% FBS. Ba/F3 cell lines were maintained in RPMI 1640 medium, supplemented with 1% Penicillin/Streptomycin solution, 10% heat-inactivated FBS and 10 ng/ml recombinant murine interleukin-3 (IL-3). Both cell lines were purchased from DSMZ (Braunschweig, Germany).

### Stable transfection of Ba/F3 cells

In order to generate stably transfected Ba/F3 cells co-expressing wild-type KIT or KIT-D816V together with SLAP2 or empty vector, the retroviral vector pMSCV was used. First, EcoPack packaging cells were transfected with pMSCVpuro-KIT-WT or pMSCVpuro-KIT-D816V using Lipofectamine 2000, and incubated for 72 hours. Supernatants containing viral particles were collected and used for infection of Ba/F3 cells. To confirm the success of transfection, cells were selected against 1.2 µg/ml puromycin and KIT expression was verified using flow cytometry and Western blotting. Packaging cells were then transfected with pMSCVneo empty vector or pMSCVneo-SLAP2-WT-FLAG vectors. Viral particles were collected from supernatants and used for further infection of Ba/F3 cells expressing either wild-type KIT or KIT-D816V. Ba/F3 cells were selected for two weeks against 0.8 mg/ml G-418 and the expression of SLAP2 was confirmed by Western blotting.

### Immunoprecipitation and Western blotting

Prior to SCF stimulation, Ba/F3 cells were washed three times with DPBS to remove cytokines and serum and maintained in RPMI 1640 medium for 4 hours at 37 °C. Ba/F3 and COS-1 cells were stimulated with 100 ng/ml SCF for the given amount of time. After stimulation, cells were immediately placed on ice, washed once with ice-cold phosphate buffered saline (PBS) and lysed for 15 min on ice in 1% Triton X-100 lysis buffer containing 1 mM Na_3_VO_4_, 1% Trasylol and 1 mM PMSF. Lysis, immunoprecipitation and western blotting methods were previously described^34^. Immunodetection was performed with Luminata Forte Western HRP Substrate and a LAS-3000 CCD camera (Fujifilm, Japan). Multi-Gauge software (Fujifilm) was used to quantify the signal intensity.

### Cell viability assays

Cells were washed three times with DPBS to remove IL-3 and then resuspended in RPMI 1640 medium supplemented with 1% Penicillin/Streptomycin solution and 10% heat-inactivated FBS. In order to measure cell viability using PrestoBlue, cells were seeded in 96-well plates at a concentration of 10,000 cells per 90 µl using three different incubation conditions: either in the presence of IL-3 or in the presence of SCF or without cytokines. After incubation for 48 hours, 10 µl of PrestoBlue was added to each well which were further incubated for 2 hours. Fluorescence was measured using a 96-well plate reader. For determination of cell viability by measuring the amount of ATP present in the cells, cells were seeded in 96-well plates at a concentration of 10,000 cells per 100 µl using three different incubation conditions: either in presence of IL-3 or in presence of SCF or without cytokines. After incubation for 48 hours, 100 µl of CellTiterGlo reagent was added to each well and luminescence was recorded using a 96-well plate reader.

### Apoptosis assay

100,000 cells/ml were seeded in 12-well plate using three different incubation conditions: either with IL-3, with SCF or without cytokines. After 48 hour incubation, cells were stained with fluorescent Annexin V and the percentage of Annexin V negative cells was analyzed using BD FACSVerse Flow cytometer (BD Biosciences, San Jose, CA, USA) and denoted as living cells.

### Clonogenic assay

Cells were washed three times using DPBS to remove cytokines. Around 500 cells were re-suspended in a 0.5 ml mixture of IMDM (20%) and methylcellulose medium (80%). Cells were seeded in 24-well plates and incubated for 7 days at 37 °C before the colonies were counted. Pictures of colonies were recorded using Infinity Analyze software (Luminera Corporation). Relative colony size was determined using ImageJ software.

### KIT degradation assay

Ba/F3-WT-KIT cells expressing either empty vector or SLAP2 were washed three times with DPBS to remove IL3, treated with 0.1 mg/ml cycloheximide and incubated for 30 min at 37 °C. Cells were then stimulated with 100 ng/ml SCF for 30 min. After stimulation, cells were immediately washed with ice-cold PBS, lysed in 1% Triton X-100 lysis buffer containing protease and phosphatase inhibitor mixture and lysates were further analyzed by Western blotting.

### Affinity fishing of SLAP2 using immobilized KIT phosphopeptides

For affinity fishing of SLAP2, synthetic phosphopeptides with following sequences (corresponding to known KIT tyrosine phosphorylation sites) were used: pY-553: CYLQKPMpYEVQWKVV, pY-568: CINGNNpYVYIDPT, pY-570: CINGNNYVpYIDPT, pY-721: CSDSTNEpYMDMKPGV, pY730: CSDSTNEYMDMKPGVSpYVVPTKA, pY-823: CKNDSNpYVVKGA, pY-900: CMLSPEHAPAEMpYDIMKT. The non-phosphorylated peptide corresponding to Y568 was used as a control. Phosphopeptides were immobilized on UltraLink beads following the manufacturer’s instructions. COS-1 cells were transfected with wild-type SLAP2 using Lipofectamine 2000. 24 hours after transfection, cells were lysed, processed and the lysates were incubated at 4 °C for 1 hour with 20 µl of 1:1 immobilized peptide beads slurry. Beads were washed four times with lysis buffer containing 500 mM NaCl and subjected to Western blot analysis.

### Statistical analysis

All statistical analysis was performed using GraphPad Prism 5.0 software. To determine statistical significance, one-way ANOVA with Bonferroni’s post-test and one-tailed Student’s t-test were used. Data is expressed as the mean ± SE. P values less than 0.05 were considered significant (*P ≤ 0.05, **P ≤ 0.01, ***P ≤ 0.001, ns: P > 0.05).
